# LncRNA XIST modulates HIF‐1A/AXL signaling pathway by inhibiting miR‐93‐5p in colorectal cancer

**DOI:** 10.1002/mgg3.1112

**Published:** 2020-02-15

**Authors:** Li‐guang Yang, Ming‐zheng Cao, Jie Zhang, Xiao‐yan Li, Qin‐li Sun

**Affiliations:** ^1^ Department of Gastrointestinal Surgery Linyi Central Hospital Linyi China

**Keywords:** colorectal cancer, HIF‐1A, LncRNA XIST, migration, miR‐93‐5p

## Abstract

**Background:**

Long noncoding RNA (LncRNA) *XIST* is one of the genes that exists in different types of cancers. Earlier researches showed that *XIST* can advance the progression of colorectal cancer. Nevertheless, the potential molecular mechanism of *XIST* in combination with miR‐93‐5p has not been explored in colorectal cancer.

**Methods:**

We performed qRT‐PCR to explore the level of *XIST*. And a serious experiments in vitro and in vivo were performed to explore the function of *XIST*. The relationship between *XIST*/*HIF‐1A* and miR‐93‐5p was confirmed by RIP and dual‐luciferase assays.

**Results:**

In the present research, our team demonstrated the upregulation of *XIST* expression, which was related to tumor progression, and the downregulation of miR‐93‐5p in cells and tissues of colorectal cancer. *XIST* is the competitive endogenous RNA of miR‐93‐5p to promote *HIF‐1A*, and then the upregulated *AXL* level facilitates the EMT process, migration, and proliferation of colorectal cancer. At last, we proved that *XIST* enhanced the in vivo and in vitro activities of colorectal cancer by regulating *AXL* signaling.

**Conclusion:**

In summary, the above results indicate that *XIST* promotes colorectal cancer tumorigenesis by regulating miR‐93‐5p/*HIF‐1A*/*AXL* signaling pathway, which will supply a novel perspective to diagnose and treat colorectal cancer disease.

## Graphical abstract

Increasing evidence suggest that lncRNA expression alterations have a vital impact in the process of biological process in cancer. However, the role of lncRNAs in colorectal cancer remains largely to be explored. Here, we report that *XIST* promotes colorectal cancer tumorigenesis by regulating miR‐93‐5p/HIF‐1A/AXL signaling pathway.

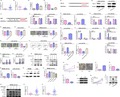

## 1 INTRODUCTION

Colorectal cancer ranks the fourth deadly cancer in the world (Hatano et al., 2017). The onset of colorectal cancer was early, the critical genetic mutation of it was regarded as important in the disease development process, and many researchers have interests in finding out whether there are specific mutations that stimulate the colon, and if there are strategies to prevent cancer (Birt & Phillips, 2014). The incidence of colorectal cancer differs between countries and are increasing across the world. The incidence rate of colorectal cancer is increasing in many countries, probably because of the extensive adoption of the Western lifestyle and diet. Epidemiological studies have showed that diets with rich fiber or vegetable and physical activity can reduce the colorectal cancer rates, and consumption of processed or red meat, alcoholic beverages, and overconsumption as reflected in obesity will increase the rates of colorectal cancer (Chan & Giovannucci, 2010; Glade, 1999). Colorectal cancer patients showed poor prognosis due to resistance to traditional therapies (Hu, Li, Gao, & Cho, 2016). Even after surgical resection and aggressive chemotherapy, 50% of colorectal carcinoma patients develop recurrent disease (Anitha, Maya, Sivaram, Mony, & Jayakumar, 2016). Thus, it is vital to explore an professional and effective way for treatment and early diagnosis of colorectal cancer.

LncRNAs belong to a various class of transcripts, which have more than 200 nucleotides, they are widely synthetized in the genome, and correlated with the development of pathology and physiology (Weidle, Birzele, Kollmorgen, & Ruger, 2017). LncRNAs could not encode protein while could modulate gene levels at the posttranscription and transcription of gene (Cui et al., 2016; Derrien et al., 2012). More and more evidence shows that lncRNAs involve the mechanisms of modulating the behavior of cancer cell, for example, the process of metastasis, proliferation, apoptosis, drug resistance, and epithelial‐mesenchymal transition (EMT) (Huang et al., 2017; Quinn & Chang, 2016; Yoshimura, Matsuda, Yamamoto, Kamiya, & Ishiwata, 2018). The lncRNA X‐inactive‐specific transcript (*XIST*) can function as a tumor suppressor gene or oncogene in various cancers (Mao et al., 2018), including hepatocellular carcinoma cell (Kong et al., 2018), non‐small cell lung cancer (Wang et al., 2017), colorectal cancer (Song et al., 2017), esophageal squamous cell carcinoma (Wu et al., 2017), prostate cancer (Alahari, Eastlack, & Alahari, 2016), bladder cancer (Xiong et al., 2017), and so on. Recent studies showed that *XIST* expression is increased in colorectal cancer cells (Sun, Zhang, & Liu, 2018), while the interactions between miR‐93‐5p and *XIST* in colorectal cancer have no previous report.

In our research, our team speculated that *XIST* may accelerate colorectal cancer progression by inhibiting miR‐93‐5p expression. Firstly, we determined the expression of miR‐93‐5p and *XIST* in tissues from patients who suffered from colorectal cancer and also in colorectal cancer cells. Moreover, the in vivo and in vitro potential mechanism of *XIST* in colorectal cancer development was analyzed. The present research may provide a novel perspective to treat colorectal cancer.

## 2 MATERIALS AND METHODS

### 2.1 Editorial Policies and Ethical Considerations

The study obtained the approval opinion from the Ethics Committee of Linyi Central Hospital.

### 2.2 Patients and samples

Thirty‐six colorectal cancer samples and their adjacent non‐tumorous samples (*n* = 36) were purchased from patients who underwent primary surgery from 2016 to 2018 in the Linyi Central Hospital. After surgery, the samples were immediately frozen at −80°C for further study. The details of the patients participated in this study are displayed in Table 1.

**Table 1:** The correlation of XIST expression with clinical parameters in patients with colorectal cancer

Clinicopathologic features	Number of cases	XIST expression		*p* value
		High (*n* = 36)	Low (*n* = 36)	
Gender				0.6353
Male	40	21	19	
Female	32	15	17	
Age				0.6368
<65	34	18	16	
≥65	38	18	20	
Tumor size				0.8130
＜5	33	16	17	
≥5	39	20	19	
TNM stages				0.0333*
Ⅰ/ⅠⅠ	37	15	24	
Ⅲ/Ⅳ	33	21	12	

* *p* < .05.

### 2.3 Cell lines

The human colorectal cancer cells (SW480 and LoVo) and normal colon epithelial cell (NCM460) were purchased from the Cell Bank of the Chinese Academy of Science. The cells were cultured in an incubator at 37°C in 5% CO_2_.

### 2.4 Construction and transfection of plasmid

*XIST* cDNA was extracted from samples of human colorectal cancer. The cDNA was cloned into the BamHI and XhoI sites of pLVX‐IRES‐Neo vector (Invitrogen, Carlsbad, CA, USA) to construct the pLVX‐*XIST* vector. Then transfected the vectors into colorectal cancer cells to package lentivirus using Lipofectamine 2000 (Invitrogen, Carlsbad, CA, USA). Lentivirus was used to infect the SW480 cells. For the construction of luciferase reporter vectors, the *HIF‐1A* 3′‐untranslated regions (*HIF‐1A* 3′‐UTR) and *XIST* cDNA segment, which contains the possible miR‐93‐5p mutant or binding sites, were amplified using PCR technique, and were transcribed to pGL3 luciferase reporter vector (Promega) at the site of the KpnI and XhoI.

*XIST* siRNA, the miR‐93‐5p mimics, and miR‐93‐5p antagomirs (miR‐93‐5p inhibitor) were synthesized by GenePharma Co. To construct si‐*XIST* vector, the self‐complementary hairpin DNA oligonucleotides were annealed and subcloned into the pEGFP‐N1 plasmid vector. Si‐NC vector functioned as the negative control. The LoVo cells were transfected with vectors by Lipofectamine 2000 to stably establish a cell line. According to the producer's protocol of using Lipofectamine 2000 to transfect cells, the stably transfected cells were cultured in a 6‐well plate. After transfection for 48 hr, western bolt or RT‐PCR was performed on the collected cells.

### 2.5 Proliferation test

To detect the proliferation of designated vector transfected cells, the Cell Counting Kit‐8 (CCK8) assay was applied. Besides, SW480 cells, which showed stable upregulation of *XIST*, were handled with BGB324 for 72 hr at the concentration of 1, 0.5, and 0.1 nM, and the vitality of cells was detected and the BGB324 is R428 (Selleck).

### 2.6 Flow cytometry detection

Propidium iodide (PI; BD Biosciences) and Annexin V‐FITC double staining were conducted to detect cell apoptosis. After 48 hr of transfection, the cells were harvested. PI and Annexin V‐FITC were added into the solution in accordance with the producer's instructions. The solutions were then tested with a FACSCalibur flow cytometer (BD).

### 2.7 The transwell analysis

After 24 hr transfection of indicated vector, 200‐μl serum‐free medium (2 × 10^4^/well) containing cells were planted in the upper chambers. Five hundred microliter medium containing 10% FBS was added to the lower chambers to induce the migration of cells. After incubation for 48 hr, 4% paraformaldehyde was used to fix the cells adhering to the lower surfaces, and after crystal violet staining, the cells were observed using a microscope.

### 2.8 Dual‐luciferase assay

About 0.3 μg of pGL3‐*XIST* (*XIST*‐WT), pGL3‐*XIST*‐MUTANT (*XIST*‐MUT), pGL3‐*HIF‐1A* (*HIF‐1A*‐WT), or pGL3‐*HIF‐1A*‐MUTANT (*HIF‐1A*‐MUT) was co‐transfected with NC or 40‐nM miR‐93‐5p mimics to colorectal cancer cells by Lipofectamine 2000. After 48 hr transfection, the activities of luciferase were determined by the Dual‐luciferase assay kit (Promega) in accordance with the producer's instruction.

### 2.9 RIP analysis

RNA‐binding protein immunoprecipitation (RIP) test was conducted using the EZ‐Magna RIP^TM^ RNA‐binding Protein Immunoprecipitation Kit (Millipore). The cells were lysed to prepare RIP lysis solution. 100‐μl of cell lysate was incubated with RIP buffer containing magnetic beads conjugated with human anti‐AGO2 antibody (1:50 dilution, Millipore) and negative control normal mouse IgG. Proteinase K buffer was applied to incubate samples and the extraction of targeted RNA was conducted for further research.

### 2.10 Tumorigenicity model building

Athymic female BALB/C nude mice aged 5‐week were purchased from the National Laboratory Animal Center. The mice were adaptive fed for 7 days when the experiment began. Experiment instructions were all authorized by the Ethics Committee of Linyi Central Hospital. To establish colorectal cancer xenograft models, 4 × 106 LoVo cells underwent stable transfection with NC or si‐XIST, and subcutaneously inoculated to the mouse at dorsal right flank. To study the in vivo mechanism of XIST, XIST was stably up regulated in SW480 cells, which were inoculated to each mouse. After inoculation, mice were orally treated with BGB324 (25 mg/kg) for 48 hr, twice per day. Starting from the 12th day since inoculation, every 3 days, the tumor diameter (mm) and volume (mm^3^) were measured, and the volume was determined by V = 0.5 × (longest diameter) × (shortest diameter)2. At day 30, tumor samples were obtained for further study.

### 2.11 Quantitative real‐time PCR (qRT‐PCR)

Total RNA was extracted using TRIzol (Invitrogen) from the tissues and cDNA was synthetized. The qPCR was conducted by Power SYBR Green (Takara). The relative gene expressions were normalized to the GAPDH by the comparative method of 2^‐ΔΔCT^. The miR‐93‐5p was amplified and detected with TaqMan Advanced MiRNA Assay Kit (Applied Biosystems) and normalized to U6.

### 2.12 Immunohistochemistry test

Neutral formalin (10%) was used to fix colorectal cancer samples, and the samples were then embedded in paraffin. The tissues were sliced to 4‐μm sections. After the sections were dewaxed, rehydrated, and antigen repaired, the sections were incubated with anti‐Ki‐67 antibody (Abcam) and HRP‐conjugated secondary antibody, and DAB was used for staining. At last, hematoxylin was used to counterstain the nuclei. Light microscopy was applied to observe the images. The scale of 0–3 (3, strongly positive; 2, moderately positive; 1, weakly positive; 0, negative) score was used to determine the staining intensity (Liu et al., 2015).

### 2.13 Western blot analysis

RIPA buffer was used to extract the proteins. The protein concentrations were detected. Thirty microgram protein was added to each lane, and after the lanes were separated, the proteins were transferred to the PVDF membranes (Millipore). TBST buffer containing 5% fat‐free milk powder was used to block the proteins and the proteins were incubated overnight at 4°C with antibodies of Vimentin, E‐cadherin, Snail, *HIF‐1A*, and *AXL*. β‐action was an internal control. *HIF‐1A* antibody was obtained from Cell signaling, whereas other antibodies were all obtained from Abcam.

### 2.14 Statistical analysis

Data were displayed as means ± *SD*. All in vitro studies were independently conducted for at least three times. One‐way ANOVA and unpaired two‐tailed Student's *t*‐test were conducted by Statistical Product and Service Solutions (SPSS) 13.0, and the in vitro and in vivo data were analyzed. *p* < .05 showed the results are statistically significant.

## 3 RESULTS

### 3.1 qRT‐PCR analysis for expression of *XIST* and miR‐93‐5p in colorectal cancer tissues and adjacent normal tissues

**Figure 1:**
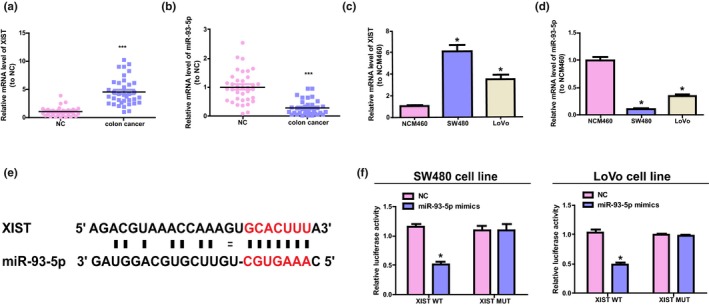
The content and relationship of miR‐93‐5p and *XIST* in colorectal cancer cells and tissues. (a, b) qRT‐PCR to detect the content of miR‐93‐5p and *XIST* in colorectal cancer tissues and adjacent normal tissues. Paired Student's *t*‐test was conducted to analyze the data. (c, d) The level of miR‐93‐5p and *XIST* was detected in normal colon epithelial cells (NCM460) and colorectal cancer cells (SW480 and LoVo). The contents of miR‐93‐5p and *XIST* were normalized to NCM460. Unpaired Student's *t*‐test was performed to analyze the discrepancies between different groups. (e) The putative binding sequence of miR‐93‐5p of the mutation sequence and wild‐type of *XIST*. (f) The results of relative luciferase tests. Unpaired Student's *t*‐test was used to perform statistical analysis. *p < .05, ***p < .001

About 36 colorectal cancer tissues and their adjacent normal tissues were obtained. *XIST* expression was detected to confirm whether *XIST* is expressed differently in different colorectal cancer samples. The results were shown in Figure 1a. The content of *XIST* was notably upregulated in colorectal cancer tissues than that of normal control. Moreover, the upregulated *XIST* expression and the TNM stage had positive association (refer to Table 1). Further, the expression of miR‐93‐5p was detected, the research displayed a reduced expression of it in colorectal cancer samples (refer to Figure 1b). Interestingly, in colorectal cancer cell lines (SW480 cell and LoVo), *XIST* level was upregulated while miR‐93‐5p was downregulated than that of normal colon epithelial cells (refer to Figure 1c,d).

### 3.2 XIST is a target of miR‐93‐5p

The bioinformatics analysis was conducted using miRcode (http://www.mircode.org). The potential relationship between miR‐93‐5p and *XIST* was described. The results showed that *XIST* has a conserved target spot of miR‐93‐5p (refer to Figure 1e). Furthermore, the activity of luciferase of pGL3‐*XIST* WT was reduced by miR‐93‐5p while the activity of pGL3‐*XIST*‐MUT remains unchanged (refer to Figure 1f). These above results show that miR‐93‐5p has a direct binding to *XIST* at the recognition sites of miRNA.

### 3.3 *XIST* modulates colorectal cancer migration, proliferation, EMT, and apoptosis by negatively regulating miR‐93‐5p expression

**Figure 2:**
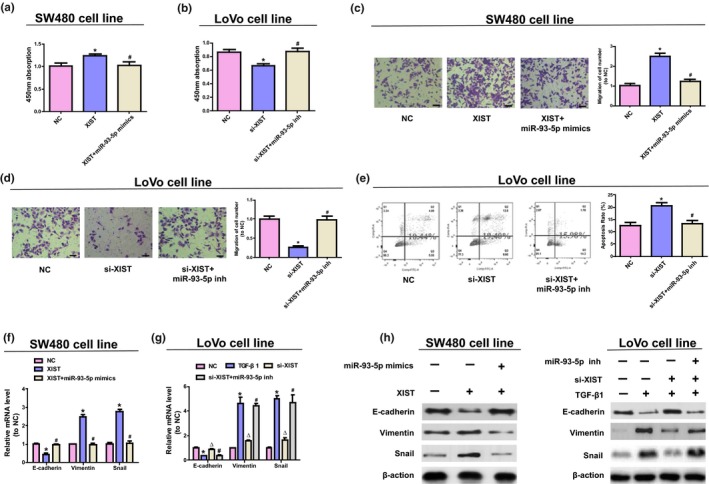
*XIST* modulates colorectal cancer migration, proliferation, EMT, and apoptosis by negatively regulating miR‐93‐5p. (a, b) Cell vitality was detected using CCK8 test in SW480 and LoVo cells. (c, d) The migration of LoVo cells and W480 cells was analyzed by Transwell assays. (e) Flow cytometry assay for LoVo cells apoptosis. (f) The mRNA level of Vimentin, Snail, and E‐cadherin in SW480 cells was detected by qRT‐PCR. (g) The expressions of EMT‐associated mRNA in LoVo cells. (h) The expressions of EMT‐associated protein in colorectal cancer cells. ^△^ *p* < .05 versus. TGF‐β1 group, *p < .05 versus. NC group, ^#^ *p* < .05 versus. si‐*XIST* or *XIST* group. One‐way ANOVA was performed to analyze data

To further investigate whether *XIST* functions through miR‐93‐5p, *XIST* expression was upregulated/downregulated and intervened with miR‐93‐5p mimics or miR‐93‐5p inhibitor in colorectal cancer cells. First of all, transfection efficiency of *XIST* overexpressing vector and *XIST* siRNA was verified by qRT‐PCR (Figure S1a,b). Transfection efficiency of miR‐93‐5p mimics and inhibitor was verified by qRT‐PCR (Figure S1c,d). The overexpression of *XIST* elevated the migration, proliferation, and the expressions in Snail and Vimentin, while reduced the level of E‐cadherin in SW480 cells; nevertheless, miR‐93‐5p mimics could ablate the above effects (refer to Figure 2a,c,f,h). In addition, downregulation of *XIST* significantly reduced the migration, proliferation, and the expression of Snail and Vimentin induced by TGF‐β1, while promoted the level of E‐cadherin and LoVo cell apoptosis, which could be inhibited by miR‐93‐5p (refer to Figure 2b,d,e,g,h). The above data indicate that *XIST* facilitates the migration, proliferation, and EMT of colorectal cancer cell, and inhibits cell apoptosis by downregulating miR‐93‐5p expression.

### 3.4 *XIST* works as the competitive endogenous RNA (ceRNA) for miR‐93‐5p and promotes *HIF‐1A* level

**Figure 3:**
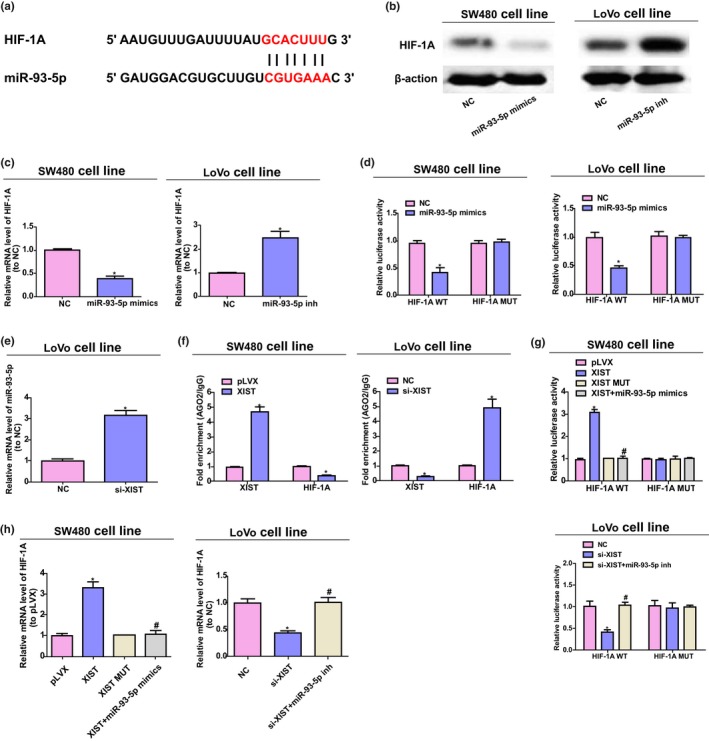
*XIST* is a ceRNA for miR‐93‐5p to promote the level of *HIF‐1A*. (a) The putative 3′UTR site for miR‐93‐5p binding of *HIF‐1A* mutation and wild‐type sequences (*HIF‐1A*‐MUT) and (*HIF‐1A*‐WT), respectively. (b, c) The mRNA and protein expressions of *HIF‐1A* in LoVo and SW480 cells. (d) Luciferase activity assay. (e) The miR‐93‐5p expression in LoVo cells transfected with NC or si‐*XIST*. Unpaired Student's *t*‐test was conducted to analyze data. (f) RIP test of the AGO2 enrichment of *HIF‐1A* and *XIST* transcripts compared with IgG of pLVX‐*XIST* or si‐*XIST* transfected cells. (g) The activity of luciferase of pGL3 reporters containing mutant *HIF‐1A* or wild‐type 3′UTR, which has indicated treatment in colorectal cancer cells. (h) The mRNA expression of *HIF‐1A* in SW480 and LoVo cells by qRT‐PCR analysis. *p < .05 versus. pLVX group, ^#^ *p* < .05 versus. *XIST* group in SW480 cells. ^#^ *p* < .05 versus. si‐*XIST* group in LoVo cells, *p < .05 versus. NC group. One‐way ANOVA was performed to analyze data

By TargetScan (http://www.targetscan.org) searching, our team discovered that miR‐93‐5p was capable of binding to 3′UTR site of *HIF‐1A* (refer to Figure 3a). Furthermore, in SW480 cells, miR‐93‐5p mimics remarkably reduced the mRNA and protein expression of *HIF‐1A*, and miR‐93‐5p downregulation increased the level of *HIF‐1A* in LoVo cells (refer to Figure 3b,c). Moreover, miR‐93‐5p remarkably attenuated the activity of luciferase of pGL3‐*HIF‐1A* WT transfected cells and did not change the activity of luciferase of pGL3‐*HIF‐1A*‐MUT transfected cells, indicating that 3′UTR site of *HIF‐1A* and miR‐93‐5p has a direct interaction (refer to Figure 3d).

*HIF‐1A* and *XIST* have common miR‐93‐5p response elements (refer to Figures 1e and 3a). Therefore, *XIST* may work as a miR‐93‐5p ceRNA and modulate the level of *HIF‐1A* in the development process of colorectal cancer. To confirm the hypothesis, the content of miR‐93‐5p was determined after *XIST* downregulation, and our team discovered that the content of miR‐93‐5p was notably upregulated by si‐*XIST* in colorectal cancer cells (refer to Figure 3e). Afterward, AGO2 underwent RIP assay. AGO2 is a critical part to RISC, namely RNA‐induced silencing complex (Tarallo et al., 2017). As Figure 3f displayed, *XIST* overexpression results in increased AGO2 enrichment while substantially decreased *HIF‐1A* transcripts enrichment. At the same time, knockdown of *XIST* showed a contrary effect. The above results suggested that XIST was capable of competing with *HIF‐1A* to combine RISC.

Furthermore, *XIST*‐mediated sequestration of miR‐93‐5p was evaluated to determine whether it was related to the *HIF‐1A* upregulation. After upregulation of *XIST*, the activity of luciferase of *HIF‐1A* wild‐type reporters was significantly promoted, while the mutant ones remain the same, and miR‐93‐5p mimics could inhibit this effect. Moreover, the *XIST* siRNA displayed a reverse effect on *HIF‐1A* luciferase activity, and miR‐93‐5p inhibition could rescue these effects (refer to Figure 3g). Further, the above results were proved at mRNA level of *HIF‐1A* (refer to Figure 3h). All in all, the above studies indicate that for miR‐93‐5p, *XIST* works as a molecular sponge to promote the level of *HIF‐1A*.

### 3.5 In vitro and in vivo, *XIST* advances colorectal cancer tumorigenesis by *HIF‐1A*/*AXL* signaling

**Figure 4:**
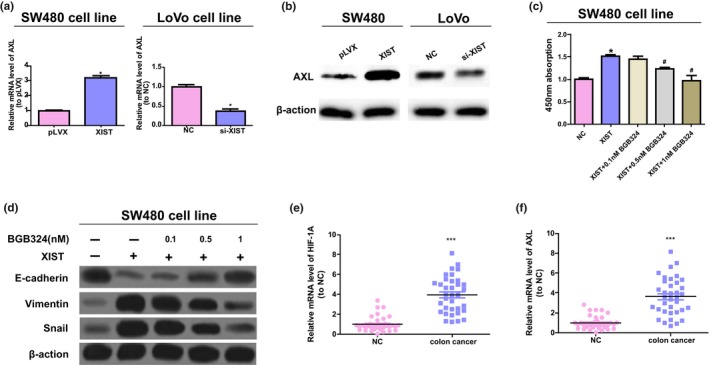
*XIST* modulates colorectal cancer activity by in vitro *AXL* signaling. (a, b) The protein and mRNA levels of *AXL* in LoVo and SW480 cells. Unpaired Student's *t*‐test was performed to analyze data. (c) *XIST* stably transfected SW480 cells were treated with BGB324 for 72 hr at the concentration of 1, 0.5, and 0.1 nM to detect the cell vitality. One‐way ANOVA was performed to analyze data. (d) The protein contents of Vimentin, Snail, and E‐cadherin in SW480 cells with stable transfection of *XIST*, and the cells were disposed with BGB324 at various concentration and detected with western blot. (e, f) The levels of *AXL* and *HIF‐1A* mRNA in paired colorectal cancer and adjacent normal samples. Paired Student's *t*‐test was conducted to analyze data. ^#^ *p* < .05 versus. *XIST* treated group, *p < .05 versus. control group. ***p < .001

**Figure 5:**
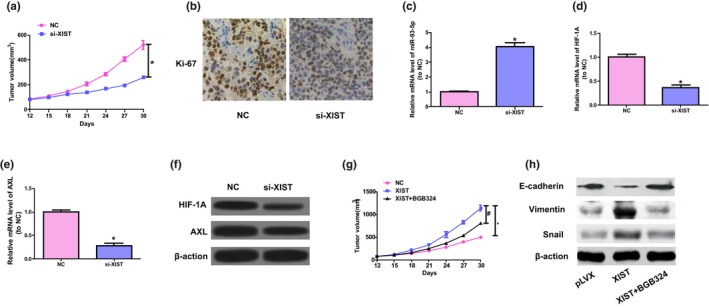
*XIST* advances cell growth of colorectal cancer by in vivo *AXL* signaling. (a) The volume of tumors was determined at the range of 12 to 30 days. (b) Representative ki‐67 staining. (c‐e) The levels of *HIF‐1A*, miR‐93‐5p, and *AXL* mRNA in tumor samples. Unpaired Student's *t*‐test was applied to conduct statistical analysis. *p < .05 versus. NC group. (f) Protein expressions of *AXL* and *HIF‐1A* in tumor tissues were detected by western blot. (g) Subcutaneous injection was performed on mice with SW480 cells of stable transfection of *XIST*; the mice orally taken BGB324 twice each day. Tumor volume was detected. (h) Western blot was conducted to detect the levels of Vimentin, E‐cadherin, and Snail. One‐way ANOVA was conducted to analyze data. ^#^ *p* < .05 versus. *XIST* group, *p < .05 versus. NC group

Previous research has shown that HIF‐1 is capable of directly binding to *AXL* and activating the expression of it (Rankin et al., 2014); therefore, to further study the mechanisms of *XIST*, the expression level of *AXL* in colorectal cancer cells was detected. Overexpression of *XIST* enhanced the protein and mRNA expressions of *AXL*, while *XIST* downregulation significantly reduced the above expression (refer to Figure 4a,b). The inhibitor BGB324 of *AXL* was used to further detect whether *XIST* enhances the activities of colorectal cancer by *AXL* signaling. The present study indicated that BGB324 inhibited the upregulation of *XIST* in the process of EMT and proliferation of SW480 cells, and the manner is dose‐related (refer to Figure 4c,d). Moreover, 36 colorectal cancer tissues and their adjacent normal tissues were obtained to determine the mRNA content of *AXL* and *HIF‐1A*. The results were displayed at Figure 4e,f, and the content of *AXL* and *HIF‐1A* mRNA of colorectal cancer samples was notably elevated than that of the normal control samples. Also, *XIST*’s function in tumorigenesis of colorectal cancer was in vivo studied*,* and the studies suggested that *XIST* knockdown dramatically inhibited the growth of tumor and the proliferation degree of antigen ki‐67 staining (refer to Figure 5a,b). The downregulation of *XIST* decreased the level of *AXL* and *HIF‐1A*, while increased miR‐93‐5p expressions (refer to Figure 5c‐f). Further, in *XIST*‐mediated colorectal cancer tumorigenesis, the function of *AXL* was further determined by orally taking BGB324, and BGB324 significantly reduced the tumor growth induced by *XIST* and the EMT process (refer to Figure 5g,h). All in all, the above results show that *XIST* enhances the tumorigenesis of colorectal cancer by the activation of *HIF‐1A*/*AXL* signaling.

## 4 DISCUSSION

The colorectal cancer represents the most frequently diagnosed cancer among both western and eastern countries, and every year, it affects more than one million individuals (Siegel, Miller, & Jemal, 2017; Yan et al., 2014). In recent years, despite the development in diagnosis and therapy of cancer has enhanced the clinical outcome, the prognosis of colorectal cancer patients is still disappointing. In the present research, the level of *XIST* was discovered to be notably elevated in colorectal cancer tissue samples than that of the adjacent normal tissues; moreover, the increased level has a positive correlation with the TNM stage (refer to Figure 1 and Table 1). Therefore, our team hypothesized that in colorectal cancer, *XIST* works as a ceRNA. The prediction program of bioinformatics was applied to confirm the results; 3’UTR of *XIST* was found to have a high conservation when binding with miR‐93‐5p. Further, our team studied the level of miR‐93‐5p and found that it showed a significant downregulation in colorectal cancer cells and tissues. Moreover, the results of dual‐luciferase reporter detection suggested that miR‐93‐5p was capable of inhibiting the activity of luciferase of pGL3‐*XIST* (refer to Figure 1). Furthermore, the overexpression of *XIST* notably elevated the process of migration, proliferation, and EMT, which were attenuated by miR‐93‐5p mimics. Reversely, *XIST* knockdown notably reduced the process of migration, proliferation, and EMT, and advanced the apoptosis process of LoVo cells, and the effects could be counteracted by inhibition of miR‐93‐5p (refer to Figure 2). In conclusion, the above results suggest that *XIST* advances the development of colorectal cancer by negatively regulating miR‐93‐5p.

The HIF proteins, especially HIF‐2α and *HIF‐1A*, are associated with the metastasis and development of tumor, and advance the epithelial‐mesenchymal transition (Yang et al., 2016). Previous studies also displayed that *HIF‐1A* was involved in the proliferation and apoptosis process of non‐small cell lung cancers (Wan & Wu, 2016). In the present study, the bioinformatics analysis results showed that *HIF‐1A* is a possible target of miR‐93‐5p. Moreover, miR‐93‐5p reduced the expression of *HIF‐1A*, which may be upregulated by the inhibition of miR‐93‐5p. Furthermore, miR‐93‐5p significantly inhibited the activity of luciferase of pGL3‐*HIF‐1A* WT. The above outcomes suggested that *HIF‐1A* directly acts on miR‐93‐5p. The RIP test results of AGO2 displayed the overexpression of *XIST* elevated the *XIST* enrichment while remarkably reduced *HIF‐1A* transcripts enrichment, while *XIST* knockdown showed an opposite effect. The above results suggested that *XIST* was capable of competing to *HIF‐1A* transcripts for RISC of AGO2‐based. Moreover, the activity of luciferase of *HIF‐1A* reporters and the content of *HIF‐1A* mRNA were elevated after the overexpression of *XIST*, but the effects could be rescued by miR‐93‐5p mimics; nevertheless, the activity of luciferase of *HIF‐1A* reporters was decreased when *XIST* was downregulated, and the effects could be reversed by miR‐93‐5p inhibition (refer to Figure 3). In conclusion, the above results indicate that *XIST* competitively combined miR‐93‐5p to increase the expression of HIF‐1A.

Earlier studies have showed that *HIF‐1A* expression is modulated by major signaling pathways, which include pathways involved in the protein kinase B (AKT) and extracellular signal‐regulated kinase (ERK) (Wan & Wu, 2016). Most studies on the AKT and ERK pathways have showed these signaling pathways play their most critical roles in the molecular signaling pathways that regulate proliferation, differentiation, growth, and survival in many, if not all, cell lines (Dent, 2013; Guo et al., 2015; Ouyang, Wang, Yan, Wang, & Lv, 2017). In the present research, in order to determine if *XIST* modulates the activities of colorectal cancer by *HIF‐1A*/*AXL* signaling, the expression of *AXL* after loss‐ and gain‐of‐function of *XIST* was detected in colorectal cancer cells. Our study showed that *XIST* overexpression significantly elevated the expression of *AXL*, and *XIST* downregulation reduced the level of *AXL*. Likewise, the expressions of *AXL* and *HIF‐1A* in colorectal cancer samples were higher than that of normal tissues. Furthermore, BGB324 suppressed the upregulation of *XIST* in the process of EMT and proliferation of SW480 cells and the manner is dose‐dependent (refer to Figure 4). Further, the knockdown of *XIST* notably inhibited ki‐67 expression and tumor growth, elevated miR‐93‐5p expression, and reduced the expressions of *AXL* and HIF‐1A, and BGB324 attenuated the effect of *XIST* in the in vivo process of EMT and tumor growth (refer to Figure 5). Nevertheless, 30 days si‐*XIST* treatment lead to a 50% reduction in the volume of tumor, while *AXL* inhibitor treatment lead to a decrease of 29%, suggesting that other *AXL*‐independent pathways played critical roles. In conclusion, the above results show that *XIST* advances tumorigenesis of colorectal cancer partly through the activation of *HIF‐1A*/*AXL* signaling.

Above all, our research showed that *XIST* is an oncogene of colorectal cancer. Increased *XIST* level has a positive correlation with the progression of tumor. *XIST* is a ceRNA for miR‐93‐5p to promote the progression of colorectal cancer partly through *HIF‐1A*/*AXL* signaling. Hence, the present study renders leading perspective to the molecular actions of *XIST* in colorectal cancer tumorigenesis, and may advance the process to diagnose lncRNA‐related disease and treatment.

## CONFLICT OF INTEREST

All authors declare no conflict of interest.

## AUTHOR CONTRIBUTIONS

Qin‐li Sun and Li‐guang Yang made substantial contributions to conception and design. Ming‐zheng Cao, Jie Zhang, and Xiao‐yan Li made acquisition of data, and performed the experiments. Li‐guang Yang and Ming‐zheng Cao wrote the draft manuscript. All authors contributed to the writing and reviewing of the manuscript, and approved the final manuscript for submission.
