# Supplementary material for: LncRNA XIST modulates HIF‐1A/AXL signaling pathway by inhibiting miR‐93‐5p in colorectal cancer
Source: Mol Genet Genomic Med. 2020 Feb 15;8(4):e1112. doi: 10.1002/mgg3.1112 (PMC7196477; doi:10.1002/mgg3.1112)
Supplement: Supplementary file 1 [file MGG3-8-e1112-s001.docx]

**Supplementary Figure Legend**

**
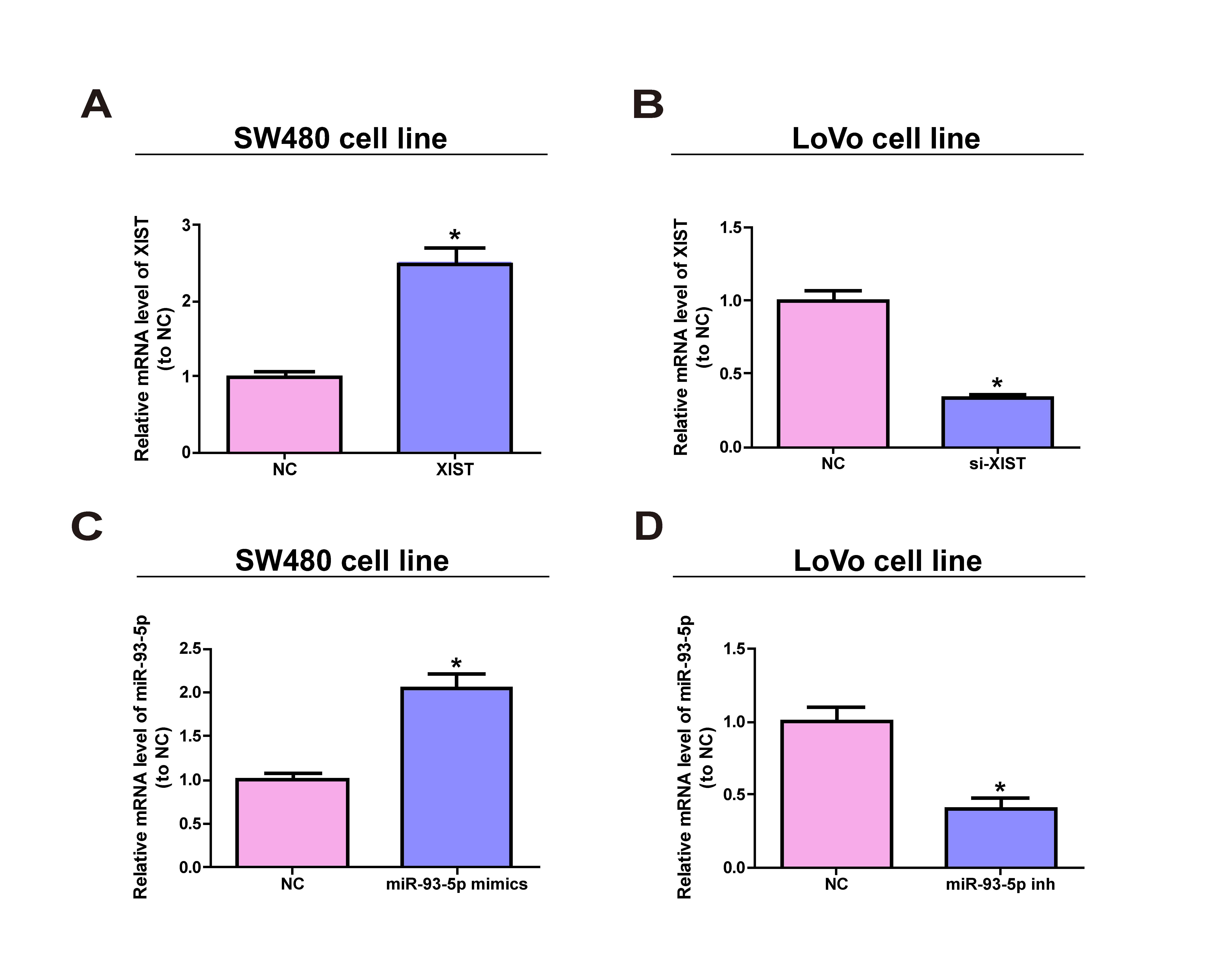
**

**Supplementary Figure 1.** (A) qRT-PCR was used to identify the effect of *XIST* in SW480 cells. (B) The mRNA level of *XIST* in LoVo cells treated with *XIST* siRNA was detected by qRT-PCR. (C) qRT-PCR was used to identify the effect of miR-93-5p mimics in SW480 cells. (D) The mRNA level of miR-93-5p was detected by qRT-PCR after transfection with miR-93-5p inhibitor in LoVo cells. Statistical analysis was conducted by unpaired Student’s t-test. *P < 0.05.
